# Multimorbidity, readmissions and mortality among older patients with potentially preventable hospitalisations: a Danish nationwide cohort study

**DOI:** 10.1007/s41999-026-01500-3

**Published:** 2026-05-19

**Authors:** Trine Worm Thøgersen, Eskild Bendix Kristiansen, Katrine Bødkergaard, Mette Geil Kollerup, Deirdre Cronin-Fenton, Marianne Lisby

**Affiliations:** 1 Research Center for Emergency Medicine, Aarhus University Hospital, Palle Juul-Jensens Boulevard 99, J103, 8200 Aarhus N, Denmark; 2 Department of Clinical Epidemiology, Aarhus University, Aarhus, Denmark; 3 Department of Clinical Medicine, Aarhus University, Aarhus, Denmark; 4 Department of Public Health, National Centre for Register-Based Research, Aarhus, Denmark; 5 University College of Northern Denmark, Hjørring, Denmark; 6 The Clinical Nursing Research Unit, Aalborg University Hospital, Aalborg, Denmark; 7 Department of Endocrinology and Internal Medicine, Aarhus University Hospital, Aarhus, Denmark

**Keywords:** Multimorbidity, Readmissions, Mortality, Ambulatory care sensitive conditions

## Abstract

**Purpose:**

We investigated the association between multimorbidity level, all-cause 30-day readmissions, and post-discharge 30-day mortality.

**Methods:**

Using Danish national registries, we identified patients aged ≥ 65 years hospitalized for ambulatory care-sensitive conditions between 2013–2018. Multimorbidity was defined as having 2–3 (moderate) or ≥ 4 (severe) of 39 chronic conditions. We estimated 30-day cumulative incidence of readmission after discharge and used Cox regression to calculate hazard ratios (HRs) for readmission and mortality.

**Results:**

Among 178,445 patients, median age was 78 years (IQR: 71–85), 59% were female. The 30-day readmission rate was 21% for those with severe multimorbidity, compared with 12% for those without multimorbidity and 16% for those with moderate multimorbidity. The hazard of 30-day readmission was elevated for both moderate (HR 1.4, 95% CI 1.4–1.5) and severe (HR 1.9, 95% CI 1.8–1.9) multimorbidity. Additionally, moderate and severe multimorbidity were associated with increased 30-day mortality (HR 1.9, 95% CI 1.7–2.1 and HR 2.6, 95% CI 2.4–2.8, respectively).

**Conclusion:**

Multimorbidity is associated with increased risk of hospital readmission and mortality, underscoring the need for improved care coordination in acute and post-discharge settings.

**Supplementary Information:**

The online version contains supplementary material available at https://doi.org/10.1007/s41999-026-01500-3.

## Key summary points

**Aim:**

To investigate the association between multimorbidity level, all-cause 30-day readmissions, and 30-day mortality in a cohort of patients hospitalised with an ambulatory care sensitive condition.

**Findings:**

Older patients with moderate or severe multimorbidity had higher 30-day readmission and mortality compared with those without multimorbidity, increasing with the level of multimorbidity.

**Message:**

Multimorbidity increases 30-day readmission and mortality risk. Identifying high-risk patients and tailoring care during and after hospitalisation is therefore crucial.

**Supplementary Information:**

The online version contains supplementary material available at https://doi.org/10.1007/s41999-026-01500-3.

## Introduction

Multimorbidity—the coexistence of two or more long-term health conditions [1]—predisposes to complications related to disease burden, reduced functional ability, frailty, decreased quality of life, and polypharmacy [2, 3]. Advanced age and multimorbidity make older individuals particularly vulnerable to adverse outcomes when admitted to emergency departments (EDs) with acute illness. Patients aged over 65 years represent an increasing proportion of ED admissions and constitute a heterogeneous group both regarding health status and reasons for admission. Furthermore, they have heightened risk of adverse outcomes, and hospital readmission [4].

Ambulatory care sensitive conditions (ACSCs) are considered an indicator of access to, or quality of primary health care services [5]. In Denmark, ACSCs encompass nine distinct medical conditions: Lower respiratory disease, fall-related fractures, urinary tract infections, dehydration, anaemia, decubitus, gastroenteritis, obstipation, and psychosocial factors [6]. These diagnostic categories are considered "potentially preventable hospitalisations" among persons aged over 65. The Danish definition of ACSCs aligns broadly with international frameworks, such as those developed by the Agency for Healthcare Research and Quality in the United States, the UK’s National Health Services, and Australia’s National Healthcare Agreement. However, variations exist in the specific conditions included and the emphasis placed on certain chronic diseases. Denmark’s approach prioritises conditions with high prevalence in its aging population, leading to differences in how preventable hospitalisations are assessed compared with international standards. These distinctions underscore the importance of context-specific research to inform targeted interventions.

Patients with ACSCs represent a particularly vulnerable population. Their clinical signs are milder and may not be detected. Non-specific symptoms of some ACSCs can lead to delayed diagnosis and treatment, increasing the severity of the conditions upon presentation at the hospital [7]. This suggests underlying complexities in the care of these patients that may stem from health behaviour, frailty, or multiple chronic conditions. These individuals are therefore susceptible to adverse outcomes, such as prolonged hospital stays, readmissions, and increased mortality [8].

Despite extensive research on the association between multimorbidity and healthcare utilization, the impact of multimorbidity on the risk of hospital readmission and mortality among older patients with an ACSC remains unclear. We therefore investigated the association between multimorbidity level and the risk of readmission and mortality in this vulnerable population. We hypothesised that the extent of multimorbidity was associated with a higher 30-day readmission risk, and higher post-discharge 30-day mortality.

## Methods

### Study design and setting

This population-based historical cohort study used routinely collected data from Danish national registries during 2013 through 2018. The Danish healthcare system is tax-funded and offers universal health care coverage for 5.8 million citizens on both primary and secondary health care levels. All Danish citizens are assigned a civil registration number, a unique personal identification number enabling individual-level linkage across Danish registries [9]. We used the Danish Civil Registration System [10], the Danish National Patient Registry (DNPR) [11], the Danish National Prescription Registry [12], and several socioeconomic registers provided by Statistics Denmark [13] (Appendix 1).

### Study population

**Fig. 1:**
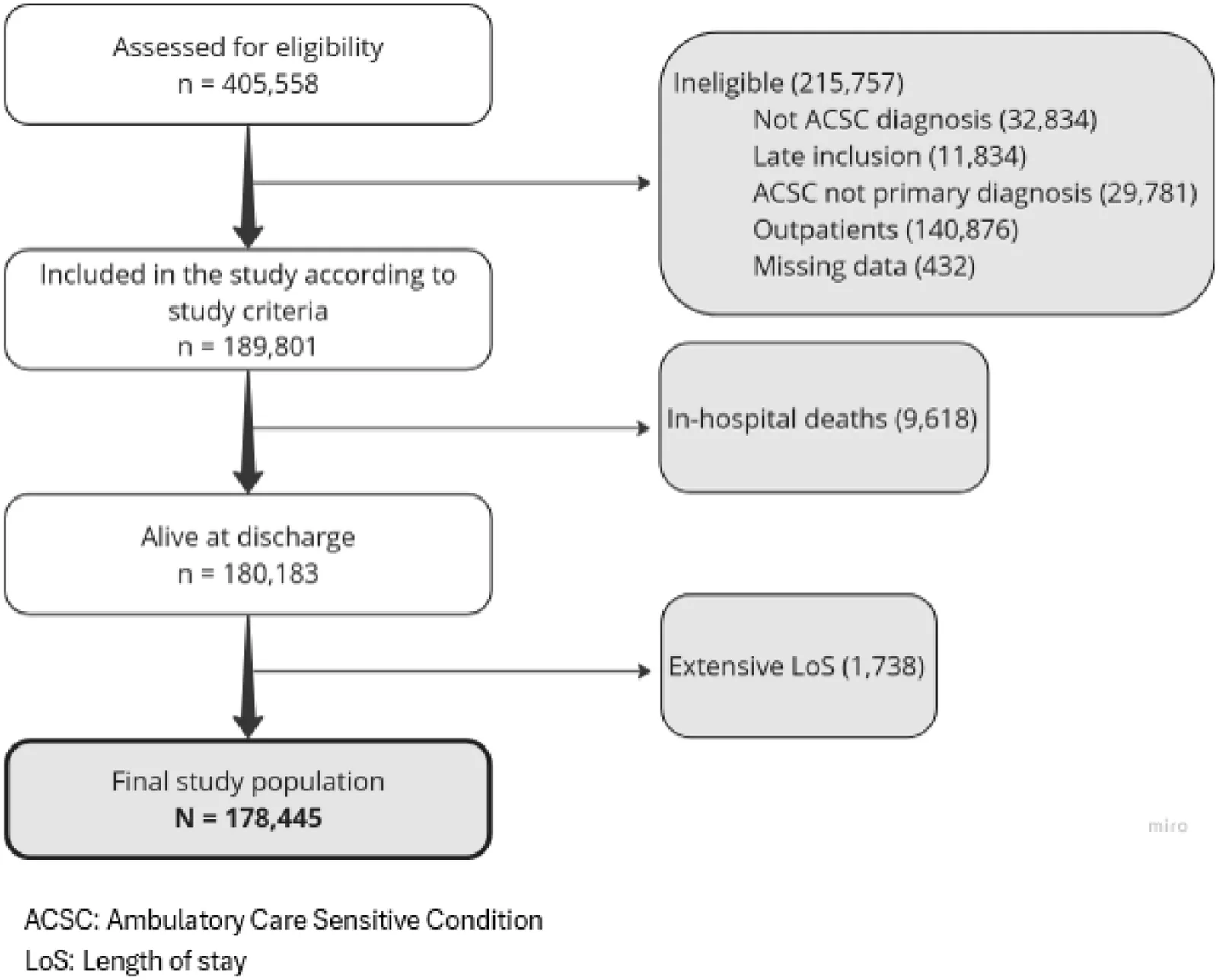
Study flowchart. Flowchart of inclusion of patients in the final cohort. A total of 405,558 patients with a hospital admission in Denmark during the inclusion period were identified. We excluded 215,757 patients who were not registered with one of the relevant ACSC diagnoses as index or where it was a secondary diagnosis. Also, 140,879 patients with outpatient status were excluded. Of inpatients with a hospital admission for an Ambulatory Care Sensitive Condition in 2013–2018, 9618 died during their admission, and 1,738 had a length of stay exceeding 30 days. In total, 178,445 patients were included in the statistical analyses

We included all patients aged 65 years or older who had an unplanned hospital admission for one of the following conditions. These admissions were identified in the DNPR and classified based on the discharge diagnosis—the main medical condition treated during their hospital stay, as recorded at discharge: "Gastroenteritis"; "Nutritional anaemia"; "Dehydration"; "Lower respiratory diseases"; "Constipation"; "Decubitus"; "Urinary tract infections"; "Fractures"; "Socioeconomic/psychosocial circumstances, care provision/medical facilities". Corresponding ICD-10 (International Classification of Diseases 10th Revision) codes are found in Appendix 2. The first relevant contact in the study period was defined as the index admission. Patients with a recorded stay of more than 30 days were excluded. A length of stay (LoS) exceeding 30 days may indicate potential classification errors, such as ongoing (open) hospital admissions, incomplete episodes (e.g., outpatient), or miscoding in the registry. Of the 180,183 patients initially identified, 1738 (1%) were excluded due to a length of stay exceeding 30 days. Patients who died during the index admission were excluded from all outcome analyses. Study flow chart is shown in Fig. 1.

### Exposure

**Fig. 2:**
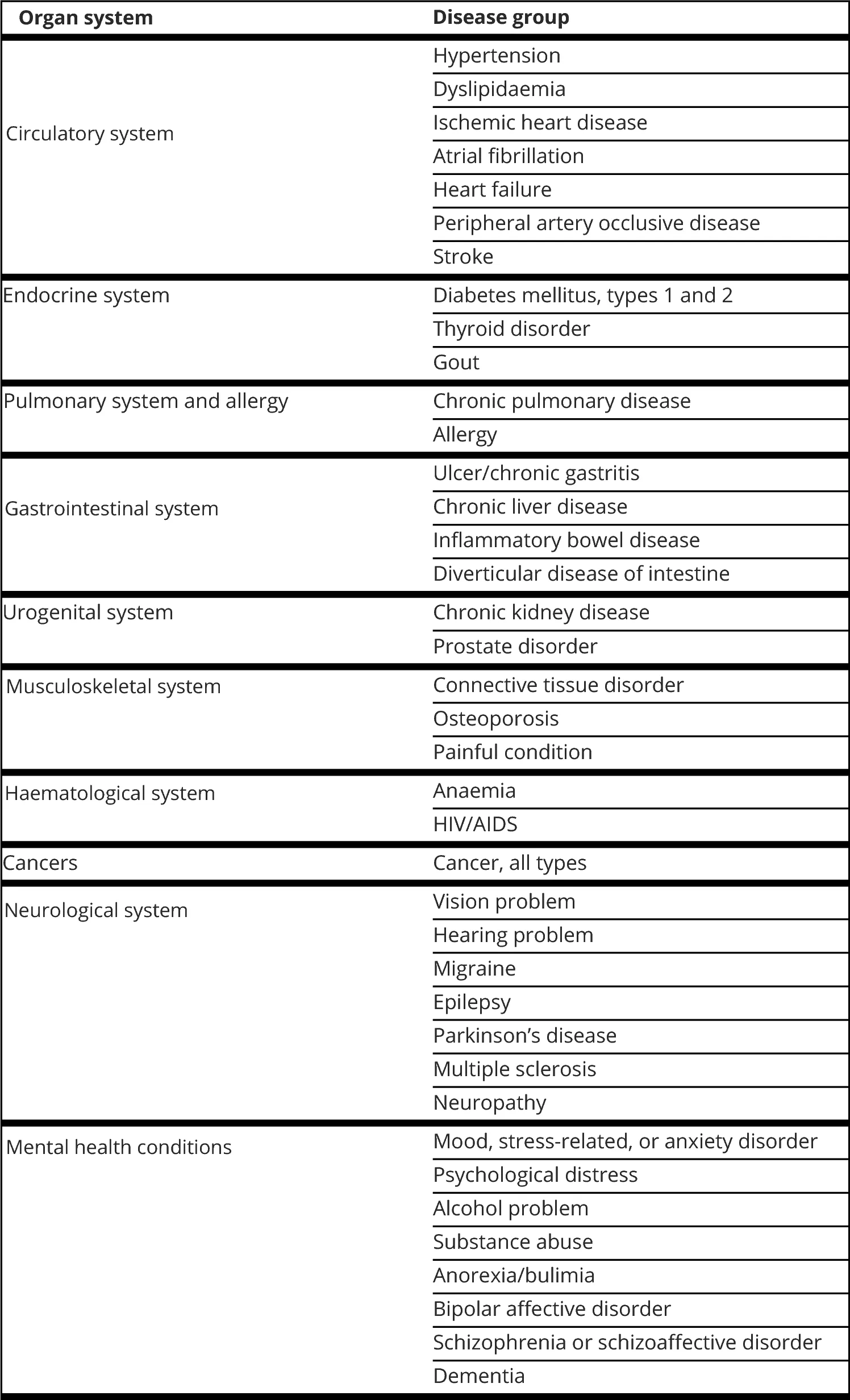
List of long-term conditions included in the multimorbidity definition according to Prior A, Fenger-Grøn M, Larsen KK, et al. Am J Epidemiol. 2016;184(3):199–210

We defined multimorbidity as having two or more of 39 specific long-term conditions covering disorders from eight organ systems, cancer, and mental health diagnoses according to the Danish Multimorbidity Index [1] (Fig. 2). Furthermore, we grouped multimorbidity into two categories: Moderate multimorbidity (2–3 disorders), and Severe multimorbidity (≥ 4 disorders). The disorders were identified by reviewing ICD-10 codes from hospital administrative systems generated in the DNPR and combining those with data on Anatomical Therapeutic Chemical (ATC) codes from prescriptions from the Danish National Prescription Registry [1].

### Covariates

From the Danish Civil Registration System, we ascertained demographic variables including age in years at index admission (continuous and in categories 65–74 years, 75–84 years, 85 years or older), sex, region of residence, cohabitation status, and civil status. From DNPR, we retrieved data on previous hospital contacts in the year preceding index admission [14]. We obtained socioeconomic and primary health care service utilisation information from Statistics Denmark: education level [15], homecare dependency, and number of general practitioner contacts (visits, telephone- and/or video consultations) within the preceding year from the index admission. See appendix 4 for detailed information about covariates.

### Outcomes

The primary outcome was 30-day hospital readmission after discharge. Secondary outcome was post-discharge 30-day all-cause mortality. Patients were followed to death, emigration, or the first event of interest within 30 days after discharge. Patients who died in-hospital were excluded from the follow-up analyses, as the focus of this study was specifically on post-discharge outcomes. This ensured that mortality data reflected only those events occurring after discharge, providing a clearer understanding of the factors associated with mortality post-hospitalisation.

### Statistical analyses

We tabulated descriptive characteristics of the study population according to multimorbidity status at index admission. We used the Aalen-Johansen method to model the 30-day risk of all-cause readmission and 30- and 90-day all-cause mortality overall and stratified by age-group, sex, homecare status, and discharge diagnosis. We used Cox proportional hazards regression to calculate the hazard ratio (HR) and associated 95% confidence interval (CI) of readmission and mortality associated with multimorbidity. Death was considered a competing risk. We adjusted for age (categorised), sex, cohabitation status, educational level, and previous hospitalisations.

To increase the generalisability of our findings and mitigate bias introduced by correlated observations, we limited our analyses to a single index admission and an associated readmission for each patient. This ensured independent observations, reducing the likelihood of overestimating our effect estimates.

## Results

The final analysis included 178,445 patients admitted for an ACSC. Among these patients 59% were women, median age was 78 (IQR: 71–85), 27% were 85 years or older (Table 1). Respiratory disease related conditions (viral/bacterial pneumonia and chronic obstructive pulmonary disease (COPD)) were the most common primary discharge diagnoses accounting for 39% of the admissions, followed by fractures (33%), dehydration (11%) and urinary tract infections (8%). The median length of stay was 3 days (IQR: 1–7). Overall, 33% of the cohort had moderate multimorbidity and 50% had severe multimorbidity. In all, 29% of the cohort had a combination of somatic and psychiatric disorder diagnoses. The most common morbidities were hypertension, dyslipidaemia, and ischemic heart disease (Appendix 7).

**Table 1:** Descriptive characteristics of 178,445 patients + 65 years with a hospital admission for an Ambulatory Sensitive Condition during 2013–2018 in Denmark

	No multimorbidity		Moderate multimorbidity	Severe multimorbidity
Number of patients, N (%)				
Total: 178,445		28,930 (16)	54,604 (31)	94,911 (53)
Female sex, N (%)		18,019 (63)	34,337 (63)	52,441 (55)
Age at index, median (Q1-Q3)		73 (68–80)	77 (71–85)	80 (73–86)
Age group, N (%)				
65–74 years old		16,693 (58)	22,162 (41)	27,520 (29)
75–84 years old		7988 (28)	18,629 (34)	37,674 (40)
≥ 85 years old		4249 (15)	13,813 (25)	29,717 (31)
Diagnosis at index admission, N (%)				
Dehydration		1598 (6)	5023 (9)	12,369 (13)
Constipation		1058 (4)	2507 (5)	4662 (5)
Lower respiratory		9818 (34)	20,262 (37)	38,768 (41)
Urinary tract infection		1531 (5)	4099 (8)	8438 (9)
Gastroenteritis		717 (3)	1453 (3)	2598 (3)
Fractures		13,841 (48)	20,082 (37)	24,494 (26)
Nutritional anaemia		202 (1)	845 (2)	2884 (3)
Decubitus		36 (< 0.5)	111 (< 0.5)	255 (< 0.5)
Psychosocial factors		129 (< 0.5)	222 (< 0.5)	443 (< 0.5)
LoS, days, median (Q1-Q3)		3 (1–6)	3 (1–7)	4 (1–7)
Category of multimorbidity, N (%)				
Psychiatric only			633 (1)	9 (0)
Somatic only			42,996 (79)	54,504 (57)
Combined conditions			10,975 (20)	40,398 (43)
Region of residence, N (%)				
Northern DK Region		2845 (10)	5720 (11)	9157 (10)
Central DK Region		5778 (20)	11,268 (21)	20,091 (21)
Region of Southern DK		5456 (19)	11,288 (21)	21,731 (23)
Capital Region of DK		9624 (33)	16,761 (31)	27,249 (29)
Region Zealand		5227 (18)	9567 (18)	16,683 (18)
Cohabiting, N (%)		16,112 (56)	26,074 (48)	41,799 (44)
Civil status, N (%)				
Unmarried		6653 (23)	11,224 (21)	18,226 (19)
Married		14,451 (50)	23,615 (43)	38,615 (41)
Widow(er)		7826 (27)	19,765 (36)	38,070 (40)
Education level, N (%)				
Basic		11,555 (40)	24,906 (46)	47,027 (50)
Upper secondary		10,245 (35)	17,923 (33)	30,162 (32)
Higher		5890 (20)	8580 (16)	12,252 (13)
Unknown/none		1240 (4)	3195 (6)	5470 (6)
Ethnicity, N (%)				
Danish		27,689 (96)	52,404 (96)	90,890 (96)
Immigrant		1204 (4)	2112 (4)	3872 (4)
Immigrant descendant		37 (< 0.5)	88 (< 0.5)	149 (< 0.5)
Home care, N (%)		3002 (10)	11,846 (22)	32,744 (35)
Health care utilisation previous year, median (Q1-Q3)				
Hospitalizations		0 (0–0)	0.0 (0–1)	0 (0–2)
GP contacts		12 (5–22)	21.0 (12–36)	35 (20–57)

Ambulatory Care Sensitive Condition: Lower respiratory disease, fall-related fractures, urinary tract infections, dehydration, anaemia, decubitus, gastroenteritis, obstipation, and psychosocial factors

Multimorbidity level: Moderate, 2–3 chronic conditions; Severe. ≥ 4 chronic conditions. Based on diagnoses reported in the National Patient Register or via prescription for medications in the National Prescription Registry, according to the Danish Multimorbidity Index

Q1–Q3 Interquartile range, *LoS* Length of stay

Among persons with moderate and severe multimorbidity, 63% and 54% were female, respectively. The proportion of individuals with a primary diagnosis of fractures was higher among those without multimorbidity compared with those with multimorbidity. The proportion of patients with lower respiratory conditions was similar across all multimorbidity levels. Of the total population, 10% of the patients without multimorbidity received homecare, whereas 35% of the patients with severe multimorbidity received homecare. The number of general practitioner contacts within the previous year was highest in the group with severe multimorbidity.

### Readmissions

**Fig. 3:**
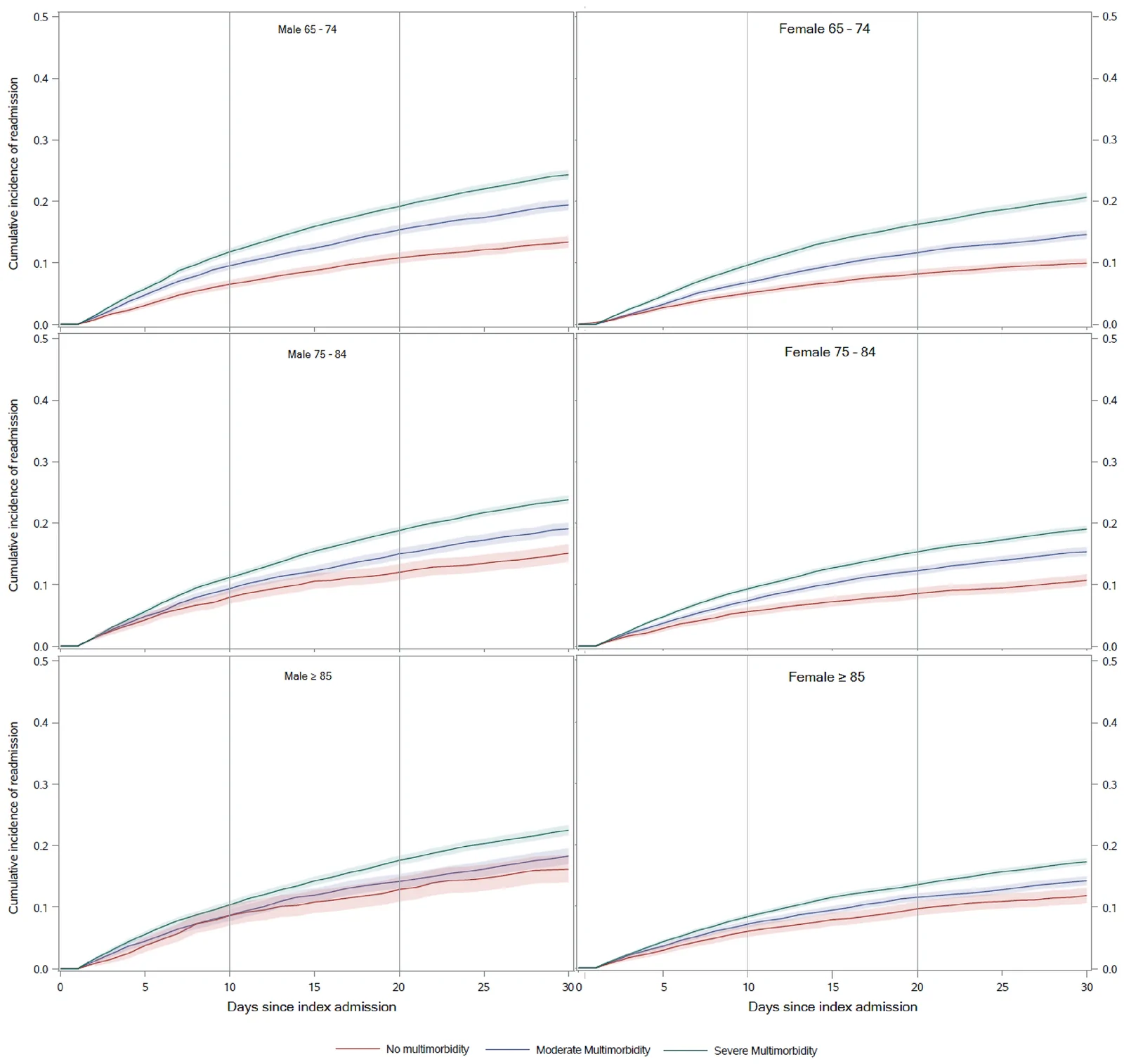
Cumulative incidence functions curves of readmissions. Cumulative incidence curves for male and female sex across three age groups. The green line shows the cumulative incidence for individuals with severe multimorbidity. The blue line shows the cumulative incidence for individuals with moderate multimorbidity. The graphs demonstrate the proportion of individuals who experienced readmissions over a 30-day follow-up period, and how the incidence of readmission varies over time across different sex and age groups

Overall, 18% of the study cohort were readmitted within 30 days after discharge. For severe multimorbidity, the proportion was 21% (Table 2). Figure 3 shows the cumulative incidence of readmission according to sex, age group and multimorbidity status. For both sexes, the difference in risk between groups was largest for the youngest age group, 65–74 years.

**Table 2:** Proportion of 30-day post-discharge readmissions and mortality among patients with a hospital admission for an Ambulatory Sensitive Condition during 2013–2018 in Denmark, according to multimorbidity level

	Total	No multimorbidity	Moderate multimorbidity	Severe multimorbidity
Number of patients	178,445 (100)	28,930 (16)	54,604 (31)	94,911 (53)
Readmission, 30 days				
Not readmitted	140,122 (79)	25,047 (87)	44,145 (81)	70,930 (75)
Readmitted	31,852 (18)	3460 (12)	8755 (16)	19,637 (21)
Competing event*	6471 (4)	423 (1)	1704 (3)	4344 (5)
Death within 30 days	9824 (6)	669 (2)	2525 (5)	6630 (7)

Estimates are shown in numbers and percentages N (%)

Ambulatory Sensitive Condition: Lower respiratory disease, fall-related fractures, urinary tract infections, dehydration, anaemia, decubitus, gastroenteritis, obstipation, and psychosocial factors

Multimorbidity level: No 0–1 chronic condition, Moderate 2–3 chronic conditions, Severe ≥ 4 chronic conditions reported in the National Patient Register or via prescription for medications in the National Prescription Registry

*Competing event: Death during hospital index admission

Compared with individuals with no multimorbidity, those with moderate and severe multimorbidity exhibited higher hazards of 30-day readmission, with adjusted hazard ratios of 1.4 (95% CI 1.4–1.5) and 1.9 (95% CI 1.8–1.9), respectively. In stratified analyses according to age group and sex, the 30-day readmission hazard remained highest for severe multimorbidity (Fig. 3).

**Fig. 4:**
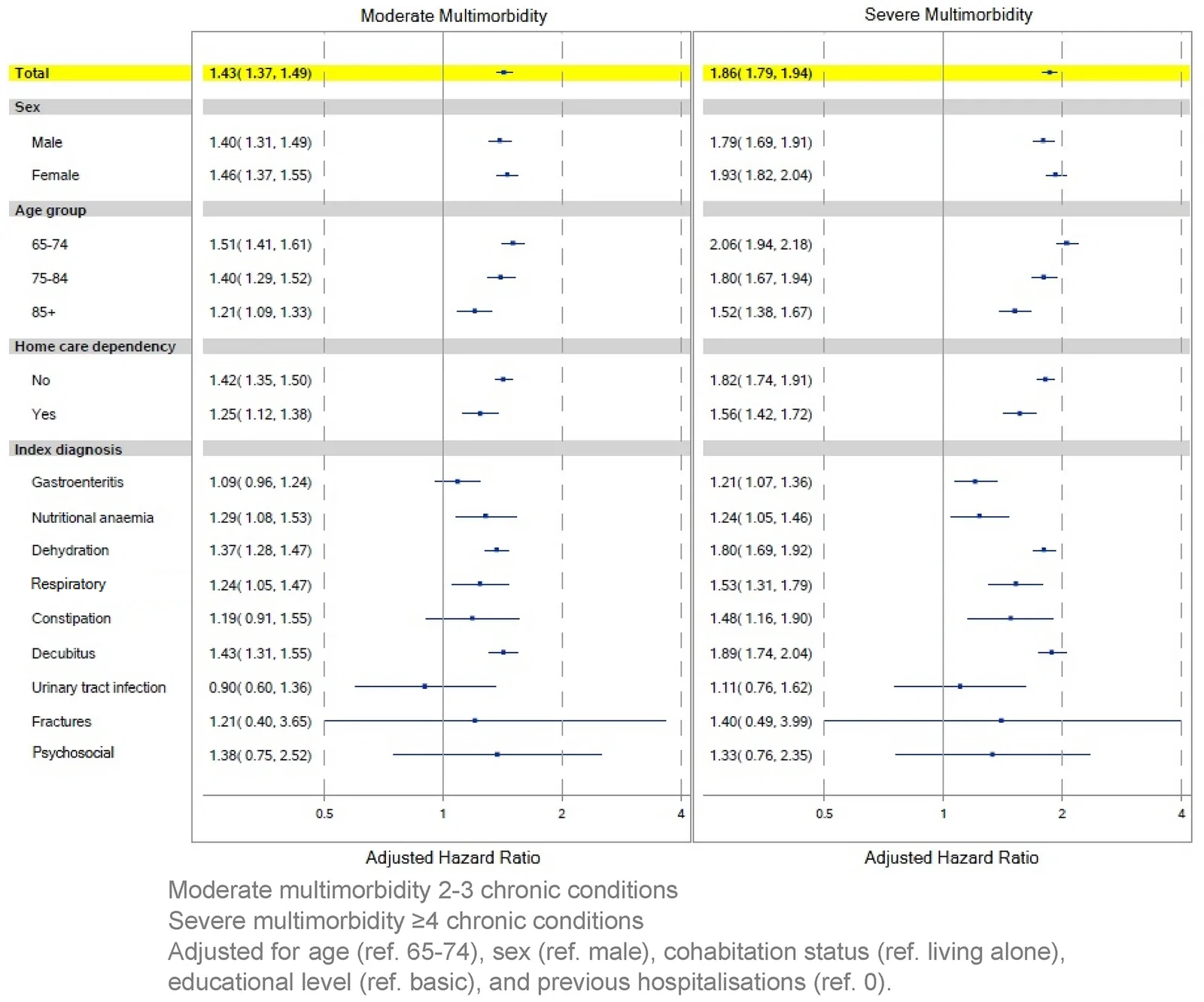
Forest plot of stratified adjusted Hazard Ratios of 30-day readmissions according to multimorbidity level. Forest plot associating moderate and severe multimorbidity with 30-day readmissions, stratified on sex, age group, home care dependency and index diagnosis. The figure shows only adjusted analyses. Results are shown for 178,445 individuals of which 54,604 (31%) had moderate multimorbidity and 94,911 (53%) had severe multimorbidity

Effect estimates varied across index diagnoses. For example, adjusted hazard ratios (aHRs) for lower respiratory diseases were 1.2 (95% CI 1.1–1.5) for moderate multimorbidity and 1.5 (95% CI 1.3–1.8) for severe multimorbidity. However, some diagnoses yielded imprecise estimates with wide confidence intervals, such as fractures (moderate: aHR 1.2, 95% CI 0.40–3.6; severe: aHR 1.4, 95% CI 0.5–4.0) (Fig. 4).

### Mortality

In total, 4% of the study cohort died during the index admission, while 6% died within the first 30 days after admission (Table 2). The adjusted 30-day mortality hazard ratios were 1.6 (95% CI 1.4–1.7) for moderate multimorbidity and 2.0 (95% CI 1.8–2.1) for severe multimorbidity (Table 3). In analyses stratified by age group and sex, the incidence rates of mortality were highest in the oldest age group, ≥ 85 years (Appendix 5).

**Table 3:** Crude and adjusted Hazard Ratios (HRs) of 30-day post-discharge readmission and mortality among patients + 65 years with a hospital admission for an Ambulatory Sensitive Condition during 2013–2018 in Denmark, according to multimorbidity level

Outcome	Moderate multimorbidity		Severe multimorbidity	
	Crude HR	Adjusted* HR	Crude HR	Adjusted* HR
30-day readmission	1.4 (1.4; 1.5)	1.4 (1.4; 1.5)	1.9 (1.8; 2.0)	1.9 (1.8; 1.9)
30-day mortality	1.9 (1.8; 2.0)	1.6 (1.5; 1.8)	2.8 (2.6; 3.0)	2.2 (2.0; 2.4)

Ambulatory Sensitive Condition*:* Lower respiratory disease, fall-related fractures, urinary tract infections, dehydration, anaemia, decubitus, gastroenteritis, obstipation, and psychosocial factors

Multimorbidity level: Moderate, 2–3 chronic conditions; Severe, ≥ 4 chronic conditions. Based on diagnoses reported in the National Patient Register or via prescription for medications in the National Prescription Registry, according to the Danish Multimorbidity Index

*Adjusted for: Age (ref. 65–74), sex (ref. male), cohabitation status (ref. living alone), educational status (ref. basic), previous hospitalisations (ref. 0)

## Discussion

This study is the first to investigate the association between multimorbidity and adverse outcomes—specifically 30-day readmission and mortality—in older adults hospitalized for ACSCs using a nationally representative Danish cohort. Our results demonstrate that moderate or severe multimorbidity is independently associated with increased risks of readmission and mortality, reinforcing its role as a key indicator of clinical complexity. These findings support the need for stratified follow-up interventions to mitigate adverse post-discharge outcomes in older adults, particularly those with complex health profiles.

The elevated risk of mortality among patients with multimorbidity is likely driven by a synergism between chronic diseases, geriatric syndromes, and frailty. While individual chronic conditions contribute directly to mortality, their cumulative and interactive effects further exacerbate vulnerability. This complexity is compounded by the fact that simple count-based multimorbidity indices do not account for disease severity, symptom burden, or duration, which are critical determinants of readmission and mortality risk. For instance, conditions such as dehydration and decubitus ulcers—often markers of underlying frailty—were associated with the highest risk estimates in our cohort. This aligns with prior research showing that frailty significantly increases mortality and readmission risks in older adults with chronic conditions, such as COPD [16] or hip fractures [17]. The overlap between multimorbidity and frailty is well-documented, with chronic diseases contributing to frailty development and frailty, in turn, amplifying the risk of multimorbidity and adverse outcomes [18].

An interesting finding was that the effect of multimorbidity on adverse outcomes was most pronounced in the 65–74 age group. This may reflect the fact that multimorbidity acts as a stronger discriminator of risk in younger-old adults, who are generally fitter and less frail than older cohorts. In this age group, the presence of multimorbidity may signal accelerated biological aging or unrecognized functional decline, making it a critical window for intervention. In contrast, among those aged 75 and older, age itself becomes a dominant risk factor, often overshadowing the incremental risk conferred by multimorbidity alone. The highest mortality incidence in our study was observed in patients aged ≥ 85 years, a group where geriatric syndromes—such as cognitive impairment, falls, and polypharmacy—frequently interact with chronic diseases to further compromise health [19]. This suggests that while multimorbidity remains important, frailty and age-related vulnerabilities may play an even larger role in determining outcomes in the oldest-old.

The Clinical Frailty Scale, now routinely used in Danish EDs as part of the DANFRAIL initiative [20, 21], provides a valuable tool for identifying high-risk patients. Frailty, as measured by the Clinical Frailty Scale strongly linked to functional dependence and adverse outcomes, including readmissions and mortality [22]. Our study underscores the need for comprehensive risk assessment that goes beyond multimorbidity counts to include frailty, geriatric syndromes, and social determinants of health. For example, over one-third of patients with severe multimorbidity in our cohort received homecare, a factor that may reflect both proactive management of complex needs and underlying vulnerabilities, such as limited social support or fragmented care coordination.

From a clinical perspective, these findings highlight several critical implications for the care of older adults with ACSCs. First, risk stratification should integrate multimorbidity indices with frailty assessments and geriatric evaluations to better capture the multidimensional nature of risk in this population. Early identification of frailty during hospitalization, combined with structured follow-up planning, has been shown to reduce readmission rates [23]. Second, interventions should be tailored to age-specific needs. For younger-old adults (65–74 years), the focus should be on preventing functional decline through medication reconciliation, chronic disease management, and physical activity programs. For older cohorts, particularly those with advanced frailty, palliative approaches may be more appropriate.

Healthcare systems must also shift from organ-specific to holistic care models that address the complex interplay of multimorbidity, frailty, and geriatric syndromes. Geriatric co-management models in EDs [19, 24] and integrated care pathways that span hospital and community settings [25] offer promising frameworks for improving outcomes. Additionally, enhanced discharge planning, including early linkage to community-based supports (e.g., home health services, meal programs), could help mitigate readmission risks. The DANFRAIL database and routine CFS screening in Danish EDs provide a strong foundation for future research, but further refinement is needed to incorporate functional trajectories, social determinants, and patient preferences into risk prediction models [26–28].

This study underscores the central role of multimorbidity in determining outcomes for older adults hospitalized with ACSCs. While frailty—often intertwined with multimorbidity—has been shown in prior research to further amplify risk, our findings highlight that multimorbidity alone is a powerful predictor of readmission and mortality, particularly in younger-old adults (65–74 years). This suggests that targeted interventions addressing chronic disease complexity in this population could yield significant benefits, even in the absence of formal frailty assessments. Moving forward, integrating comprehensive geriatric principles—such as medication optimization, functional support, and coordinated care transitions—into routine management may help mitigate adverse outcomes and improve long-term prognosis for these vulnerable patients.

### Limitations

This study utilises nationwide data with complete follow-up and high validity, reducing random error and selection bias. By integrating data from the DNPR and the Danish National Prescription Registry, we enhance the accuracy of multimorbidity identification. Although we adjusted for sociodemographic factors and prior hospital admissions as proxies for medical complexity, residual confounding may still exist due to unmeasured factors such as physical function, nutritional status, and cognitive function, which are known to be important predictors. The discrepancy between crude and adjusted hazard ratios for mortality further indicates the presence of unaccounted confounders.

The 30-day mortality outcome in this study reflects only those who survived the index admission and subsequently died within 30 days of discharge. Consequently, the reported mortality rates may be conservative estimates, particularly for conditions where in-hospital mortality is high (e.g., severe exacerbations of COPD or pneumonia).

This study utilized data from 2013 to 2018, a period selected for two key reasons. First, 2013 marked the beginning of systematic use of the ACSC indicator in Denmark, ensuring consistent and reliable identification of relevant hospital admissions. Second, from 2019 onward, the DNPR introduced new registration practices, including changes in coding and data structure. These changes could introduce potential sources of error or bias, such as inconsistencies in diagnostic coding or shifts in how admissions were classified. By restricting the study period to 2013–2018, we aimed to ensure data homogeneity and minimize the risk of confounding due to evolving registration practices. Additionally, changes in clinical guidelines, treatments, and healthcare reorganization, such as the expansion of telemedicine and cross-sectoral collaboration since our study period may have influenced readmission and mortality rates. Thus, the generalizability of our findings should be interpreted considering the evolving healthcare landscape. Furthermore, while the conceptual framework of ACSCs as a quality or performance indicator is widely recognized, the specific conditions included, and their operationalisation vary significantly across countries. This variability, along with differences in multimorbidity definitions, population characteristics (e.g., age and sex distribution), and primary diagnoses of index admissions, may impact the broader applicability of our results. Systemic challenges persist, as the Danish registries lack systematic validation for certain variables, potentially introducing information bias, particularly for patients with complex or prolonged hospitalizations.

## Conclusion

Older adults hospitalized for ACSC represent a population for whom admissions might be prevented through timely, coordinated interventions. In this study of patients aged 65 and older acutely hospitalized due to an ACSC, we found that moderate and severe multimorbidity were associated with a substantially increased risk of 30-day readmission. Similarly, there was a strong positive association between 30-day mortality and both moderate and severe multimorbidity. Early identification of high-risk individuals is critical, and along with identification of multimorbidity, structured screening in EDs and primary care settings focusing on cognitive impairment, functional limitations, and social vulnerabilities, could enhance risk stratification and guide targeted interventions. Ultimately, our results call for healthcare models that prioritize comprehensive, patient-centred care for older adults with multimorbidity, to address their complex needs and prevent avoidable hospitalization.

## Supplementary Information

Below is the link to the electronic supplementary material.

Supplementary file1 (PDF 597 KB)

## Data Availability

Due to Danish legislation, our approvals for accessing Danish data sources do not permit us to share or distribute patient data directly to third parties. Researchers interested in accessing the data can apply through the Research Service at the Danish Health Data Authority.
