# Supplementary material for: Multimorbidity, readmissions and mortality among older patients with potentially preventable hospitalisations: a Danish nationwide cohort study
Source: Eur Geriatr Med. 2026 May 19;17(4):1945–55. doi: 10.1007/s41999-026-01500-3 (PMC13562174; doi:10.1007/s41999-026-01500-3)
Supplement: Supplementary file 1 [file 41999_2026_1500_MOESM1_ESM.pdf]

Multimorbidity, readmissions and mortality among older patients with Ambulatory Care Sensitive Conditions: A Danish nationwide cohort study

Journal: European Geriatric Medicine
Corresponding Author: Trine Worm Thøgersen; Research Center for Emergency Medicine, Aarhus University Hospital, Department of Clinical Medicine, Aarhus University; trthoe@rm.dk
Co-authors: Eskild Bendix Kristiansen, Katrine Bødkergaard Nielsen, Mette Geil Kollerup, Deirdre Cronin Fenton, Marianne Lisby

Supplementary material

Appendix 1. Description of the databases included 2
Appendix 2. Diagnoses related to ambulatory care sensitive conditions 3
Appendix 3. Multimorbidity definition and identification 5
Appendix 4. Description of covariates 7
Appendix 5. Cumulative incidence function curves of mortality 8
Appendix 6. Length of stay 10
Appendix 7. Top 15 morbidities at baseline 11
Appendix 8. Sensitivity analyses 12

Appendix 1. Description of the databases included

**Registry**	**Description**
Danish Civil Registration System (CRS)<br><br>Schmidt M, Pedersen L, Sørensen HT. **The Danish Civil Registration System as a tool in epidemiology**. Eur J Epidemiol. 2014;29(8):541-9.	An administrative register established in 1968. It contains individual-level information on all persons residing in Denmark.<br><br>A unique ten-digit Civil Personal Register number assigned to all persons in the CRS allows for technically easy, cost-effective, and unambiguous individual-level record linkage of Danish registers.<br><br>Daily updated information on migration and vital status allows for nationwide cohort studies with virtually complete long-term follow-up on emigration and death.
Danish National Patient Registry (DNPR)<br><br>Schmidt M, Schmidt SA, Sandegaard JL, Ehrenstein V, Pedersen L, Sørensen HT. **The Danish National Patient Registry: a review of content, data quality, and research potential.** Clin Epidemiol. 2015;7:449-90.	A population-based administrative registry with complete nationwide coverage since 1978.<br><br>Computerized Patient Administrative Systems Information reported to the DNPR includes administrative data, diagnoses, treatments, and examinations.<br><br>DNPR data are updated continuously.
Danish National Prescription Registry<br><br>Kildemoes HW, Sørensen HT, Hallas J. **The Danish National Prescription Registry.** Scand J Public Health. 2011;39(7 Suppl):38-41.	Contains information on dispensed prescriptions, including variables at the level of the drug user, the prescriber, and the pharmacy.<br><br>Reimbursement-driven record keeping, with automated bar-code-based data entry provides data of high quality, including detailed information on the dispensed drug.
Statistics Denmark<br><br>https://www.dst.dk/en/TilSalg/Forskningsservice/Dataadgang [Danish]	Statistics Denmark has compiled a wide range of historical register data in a main database within Research Services.<br><br>The registers are updated at least once a year.

Appendix 2. Diagnoses related to ambulatory care sensitive conditions

**Diagnosis group**	**ICD-10 codes**	**Description**
Dehydration	(E869)	Volume depletion, unspecified
Constipation	(K590)	Constipation
Respiratory diseases	(J12-15, J18, J20-22, J40-47)	Viral pneumonia<br>Pneumonia due to Streptococcus pneumonia<br>Pneumonia due to Hemophilus influenza<br>Bacterial pneumonia<br>Pneumonia, unspecified organism<br><br>Other acute lower respiratory infections<br>Chronic lower respiratory diseases<br><br>Bronchitis, not specified as acute or chronic<br>Simple and mucopurulent chronic bronchitis<br>Unspecified chronic bronchitis<br>Emphysema<br>Other chronic obstructive pulmonary disease<br>Asthma<br>Bronchiectasis<br>Chronic lung allograft dysfunction
Urinary tract infections	(N30, excluding N303 and N304)	Cystitis<br>(excluding Trigonitis and Irradiation cystitis)
Gastroenteritis	(A09)	Infectious gastroenteritis and colitis, unspecified
Fractures	(S02, S12, S22, S32, S42, S52, S62, S72, S82, S92)	Fracture of skull and facial bones<br>Fracture of cervical vertebra and other parts of neck<br>Fracture of rib(s), sternum and thoracic spine<br>Fracture of lumbar spine and pelvis<br>Fracture of shoulder and upper arm<br>Fracture of forearm<br>Fracture at wrist and hand level<br>Fracture of femur<br>Fracture of lower leg, including ankle<br>Fracture of foot and toe, except ankle
Nutritional anaemia	(D50-53)	Nutritional anaemias
Decubitus	(L89)	Pressure ulcer

Psychosocial circumstances	(Z59, Z74, Z75)	Problems related to housing and economic circumstances<br>Problems related to care provider dependency<br>Problems related to medical facilities and other health care

Appendix 3. Multimorbidity definition and identification

The definition and identification of multimorbidity was based on the algorithm below adapted from *Prior A, Fenger-Grøn M, Larsen KK, Larsen FB, Robinson KM, Nielsen MG, et al. The Association Between Perceived Stress and Mortality Among People With Multimorbidity: A Prospective Population-Based Cohort Study. Am J Epidemiol. 2016;184(3):199-210.*

From national health registries, the disorders were identified by reviewing ICD-10 codes (International Classification of Diseases 10th Revision) from hospital administrative systems generated in the Danish National Patient Registry (DNPR) and combining those with data on Anatomical Therapeutic Chemical (ATC) codes from prescriptions from the Danish National Prescription Registry.

We grouped multimorbidity into three categories: No multimorbidity (0-1 disorders), Moderate multimorbidity (2-3 disorders), and Severe multimorbidity (≥4 disorders).

**Organ system**	**Disease group**	**ICD-10 codes** recorded in DNPR<br><br>*ever<br>**within last two years<br>***within last five years	**ATC-code drug** redeemed prescription<br><br>* twice last year<br>** four times last year
**Circulatory system**			
	Hypertension	I10-I13, I15*	C02, C04, C07, C08, C09, C03*
	Dyslipidaemia	E78**	C10*
	Ischemic heart disease	I20-I25*	C01DA*
	Atrial fibrillation	I48*	
	Heart failure	I50*	
	Peripheral artery occlusive disease	I70-I74*	
	Stroke	I60-I64, I69*	
**Endocrine system**			
	Diabetes mellitus, types 1 and 2	E10-E14*	A10A, A10B*
	Thyroid disorder	E00-E05, E061-E069, E07**	H03*
	Gout	E79, M10*	
**Pulmonary system and allergy**			
	Chronic pulmonary disease		R03*

	Allergy		R06AX, R06AE07, R06AE09, R01AC, R01AD*
**Gastrointestinal system**			
	Ulcer/chronic gastritis	K221, K25-K28, K293-K295*	
	Chronic liver disease	B16-B19, K70-K74, K766, I85*	
	Inflammatory bowel disease	K50-K51*	
	Diverticular disease of intestine	K57*	
**Urogenital system**			
	Chronic kidney disease	N03, N11, N18-N19*	
	Prostate disorder	N40*	C02CA, G04C*
**Musculoskeletal system**			
	Connective tissue disorder	M05-M06, M08-M09, M30-M36, D86*	
	Osteoporosis	M80-M82*	M05B, G03XC01, H05AA*
	Painful condition		N02A, N02BA51,N02BE**
**Haematological system**			
	Anaemia	D50-D53, D55-D59, D60-D61, D63-D64**	
	HIV/AIDS	B20-B24*	
**Cancers**			
	Cancer	C00-C43, C45-C97 ***	
**Neurological system**			
	Vision problem	H40, H25, H54*	
	Hearing problem	H90-H91, H931*	
	Migraine	G43**	N02C*
	Epilepsy	G40-G47*	N03*
	Parkinson's disease	G20-G22*	
	Multiple sclerosis	G35*	
	Neuropathy	G50-G64**	
**Mental health conditions**			
	Mood, stress-related, or anxiety disorder	F32-F34, F40-F48**	
	Psychological distress		N06A*
	Alcohol problem	F101-F109**	
	Substance abuse	F11-F16, F18-F19**	
	Anorexia/bulimia	F50**	
	Bipolar affective disorder	F30-F31*	
	Schizophrenia or schizoaffective disorder	F20, F25*	
	Dementia	F00-F03, F051, G30*	N06D*

Appendix 4. Description of covariates

	Covariates	Type	Description
	CPR number	ID	Central Person Register identification number
	age in years at index admission	continuous and in categories	65-74 years, 75-84 years, 85 years or older
	sex	binary	Male, female
	region of residence	categories	the Capital Region of Denmark, Region Zealand, the Region of Southern Denmark, the Central Denmark Region, and the Northern Denmark Region
	cohabitation status categories	binary	living alone or with someone
	civil status	categories	Married, unmarried, widow(er)
	previous hospital contacts	continuous	Inpatient contacts in the year preceding index admission
	education level	categories	basic, upper secondary, higher – according to the International Standard Classification of Education (ISCED)
	ethnicity	categories	Danish, immigrant, immigrant descendant
	home care dependency	binary	defined as receiving either personal care, or practical aid, or both within the preceding year from the index admission
	home nursing	binary	Receiving nursing services from the municipality year from the index admission
	preventive home visit	binary	whether the patient had received a home visit (in-home visit with individualised assessment of functional level)<br>Home visits are a specific preventive effort provided by municipalities aiming to identify the need for individual assistance and future support.
	number of general practitioner contacts	continuous	visits, telephone and/or video consultations within the preceding year from the index admission.

Appendix 5. Cumulative incidence function curves of mortality

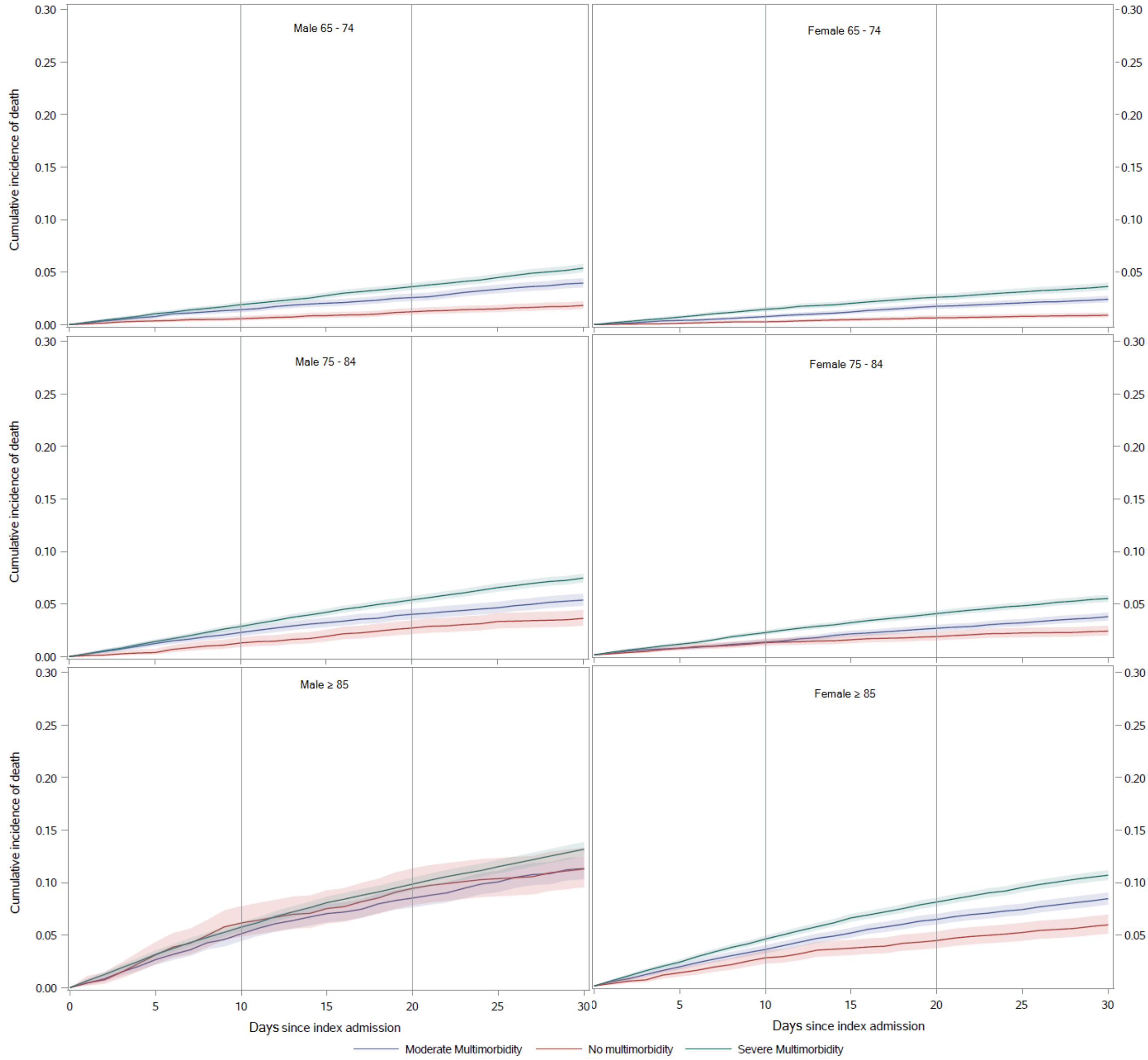

Cumulative incidence function curves of 30-day mortality according to age group and sex

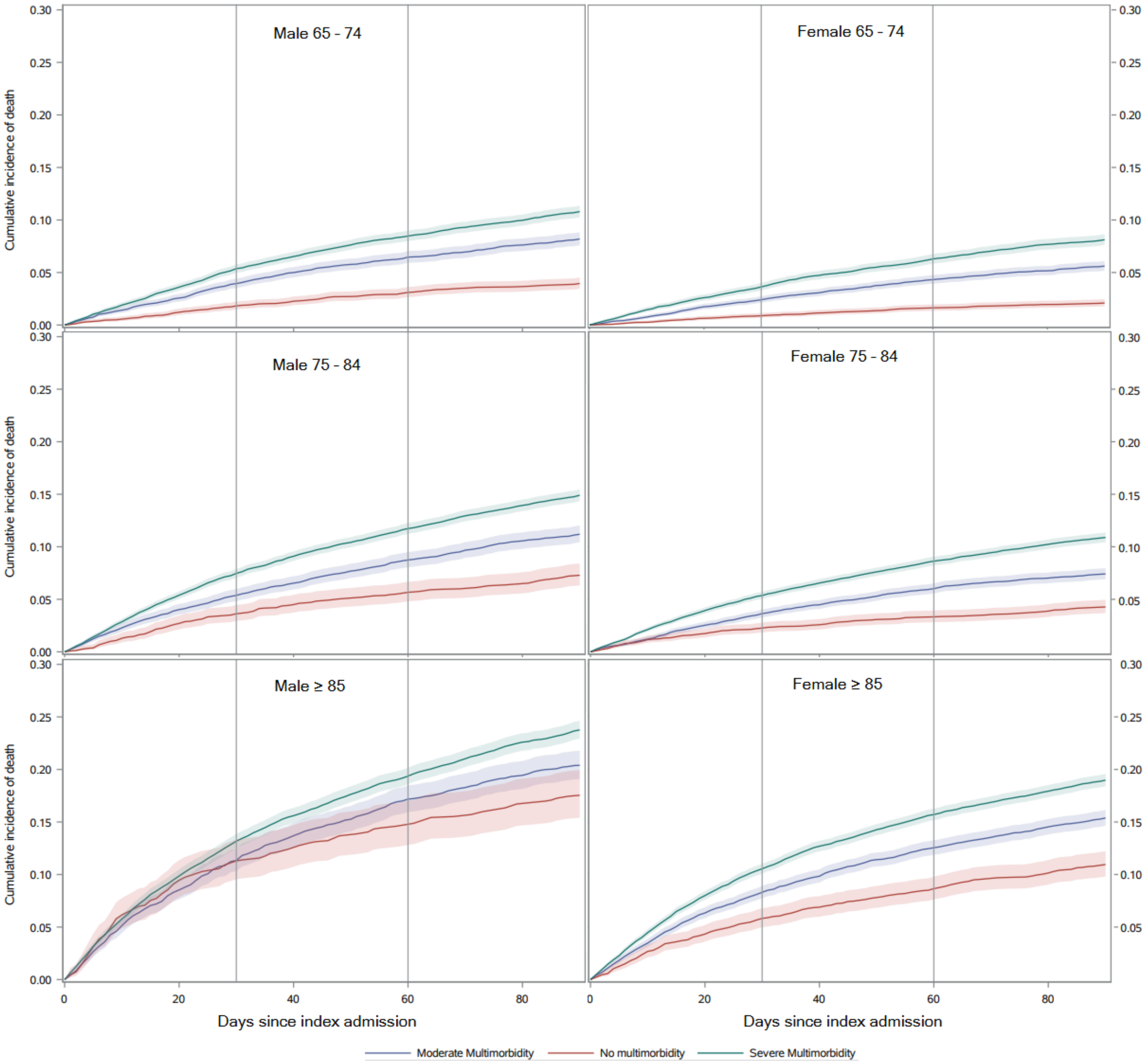

Cumulative incidence function curves of 90-day mortality according to age group and sex

Appendix 6. Length of stay

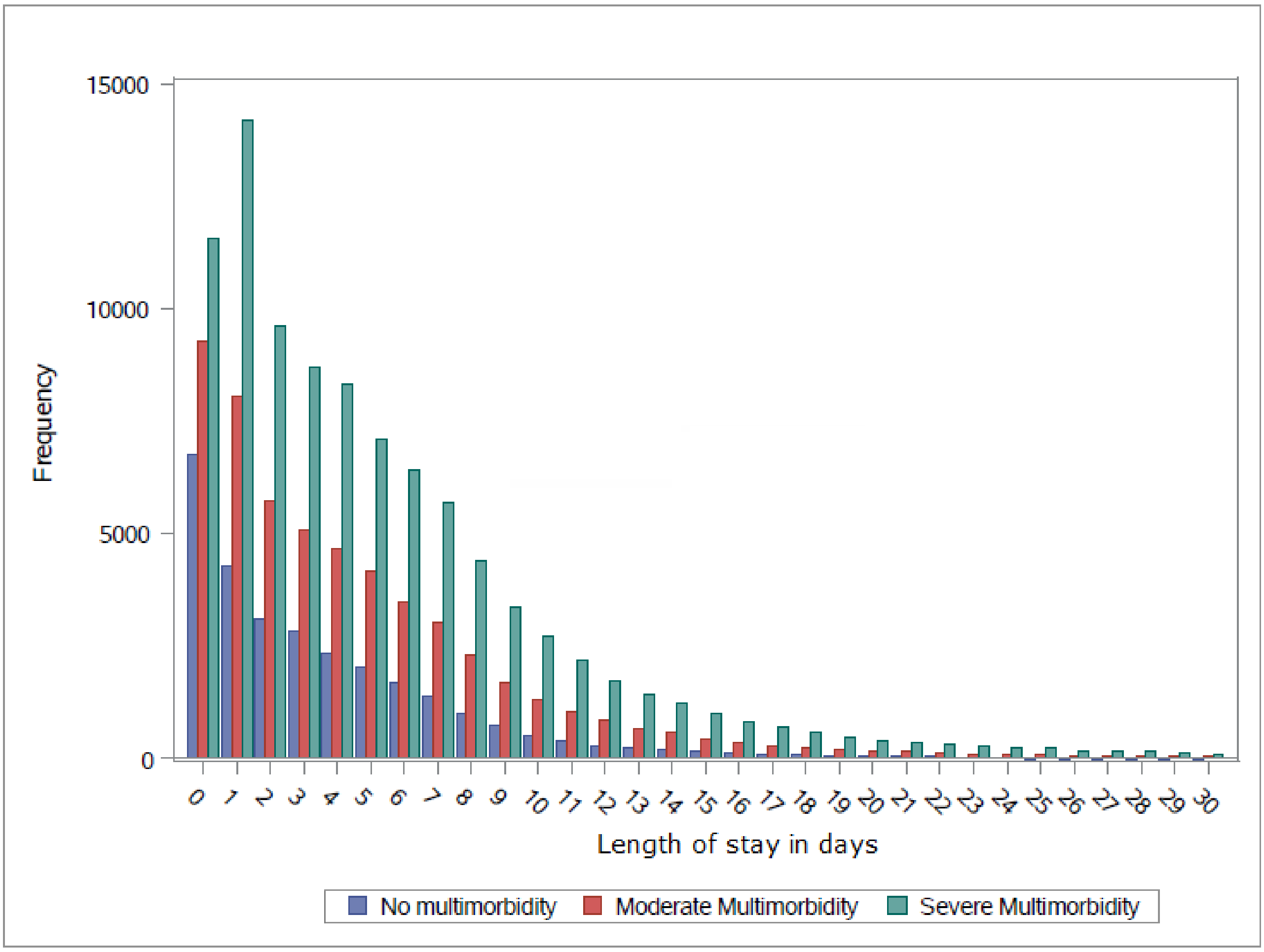

Distribution of length of stay in days according to multimorbidity level.

Appendix 7. Top 15 morbidities at baseline

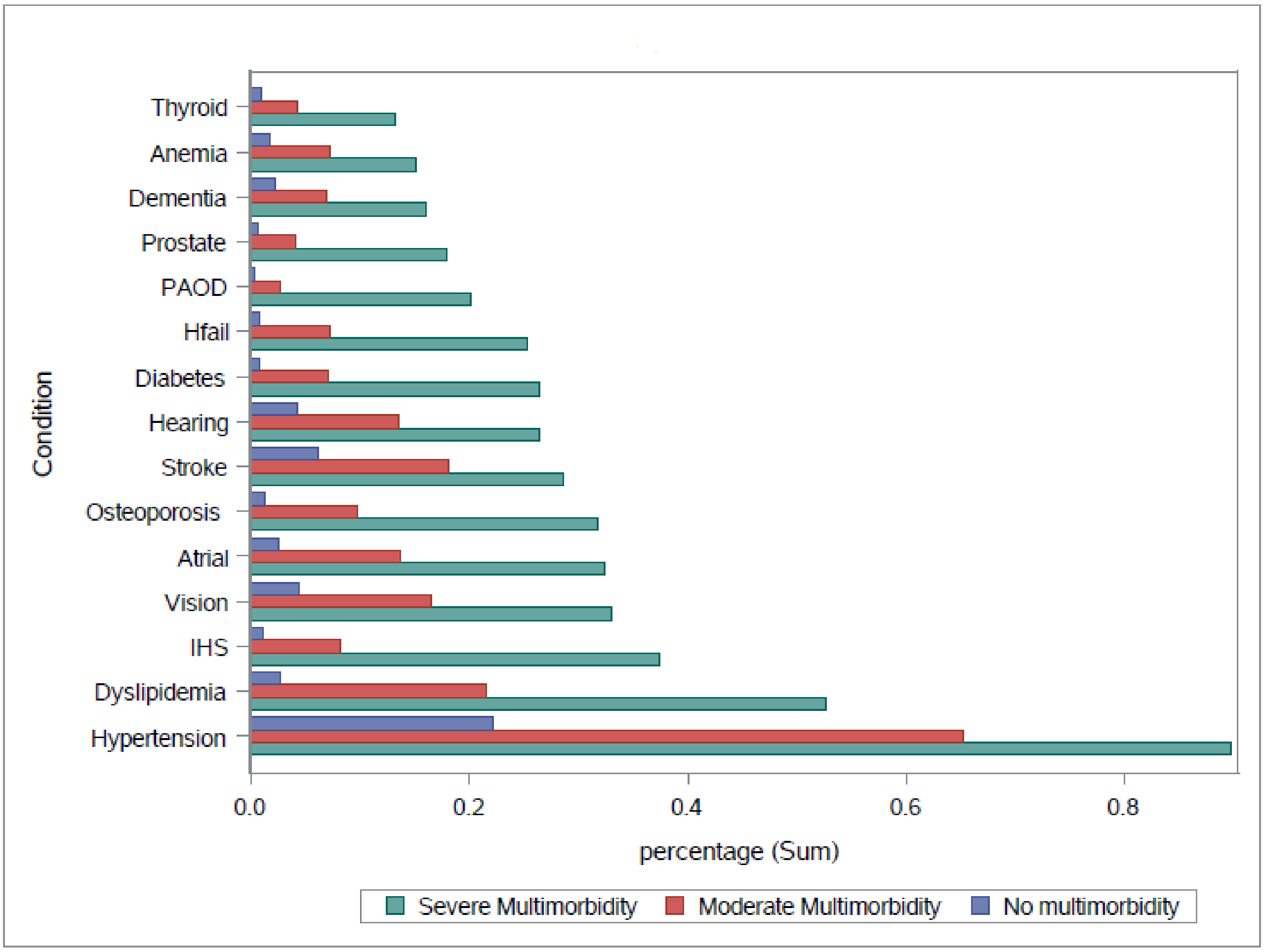

Disorders were identified by reviewing ICD-10 codes (International Classification of Diseases 10th Revision) from hospital administrative systems generated in the Danish National Patient Registry and combining those with data on Anatomical Therapeutic Chemical (ATC) codes from prescriptions from the Danish National Prescription Registry (see Appendix 3).

PAOD: Peripheral Arterial Occlusive Disease
Hfail: Congestive Heart Failure
Hearing: Hearing problem
Atrial: Atrial fibrillation
Vision: Vision problem
IHS: Ischemic heart disease

Appendix 8. Sensitivity analyses

We performed several pre-planned sensitivity analyses.

First, we repeated the primary analyses excluding hypertension from the multimorbidity index. Hypertension is a highly prevalent chronic condition in the population of persons over 65 years, and even though there are several complications related to the condition, it can vary widely in severity. We also computed an analysis in which patients with cancer diagnoses were excluded. In Denmark, patients with cancer diagnoses will often have alternative routes in the emergency care system compared with patients with other chronic conditions. Furthermore, cancers are a diverse group of diseases with varying prognoses. Some cancers, especially those with advanced stage, have high mortality rates and high readmission rates.

The results from the sensitivity analyses are shown in the two tables below. When we removed the cancer diagnoses from the multimorbidity index, the percentage of patients with severe multimorbidity decreased from 53 to 50. When we excluded the hypertension diagnosis from the multimorbidity index, the percentage of patients with severe multimorbidity decreased from 53 to 41.

Sensitivity analysis: Excluding **cancers** from the multimorbidity index	**No Multimorbidity**	**Moderate Multimorbidity**	**Severe Multimorbidity**
**Number of patients**	32,812 (18)	56,343 (32)	91,028 (50)
**Readmission within 30 days, N (%):**			
Not readmitted	27,655 (84)	45,238 (80)	68,322 (75)
Readmitted	4,500 (14)	9,245 (16)	18,600 (20)
Competing event (death)	657 (2)	1,860 (3)	4,103 (5)
**Death within 30 days, N (%)**	1,041 (3)	2,766 (5)	3,237 (7)
**Death within 90 days, N (%)**	2,097 (6)	5,445 (10)	12,044 (13)

Sensitivity analysis: Excluding **hypertension** from the multimorbidity index	**No multimorbidity**	**Moderate Multimorbidity**	**Severe Multimorbidity**
**Number of patients**	43,850 (24)	63,531 (36)	72,802 (41)
**Readmission within 30 days, N (%):**			
Not readmitted	37,502 (86)	50,205 (79)	43,508 (74)
Readmitted	5,562 (13)	11,013 (17)	15,770 (22)
Competing event (death)	786 (2)	2,313 (4)	3,524 (5)
**Death within 30 days, N (%)**	1,211 (3)	3,400 (5)	5,433 (8)

		Adjusted* HR<br>30-day readmission	Adjusted* HR<br>30-day mortality
**Multimorbidity including all 39 conditions**	Moderate Multimorbidity	1.4 (1.4 ; 1.5)	1.6 (1.5 ; 1.8)
	Severe Multimorbidity	1.9 (1.8 ; 1.9)	2.2 (2.0 ; 2.4)
**Multimorbidity excluding hypertension**	Moderate Multimorbidity	1.4 (1.4 ;1.5)	1.6 (1.5 ; 1.7)
	Severe Multimorbidity	1.8 (1.8 ; 1.9)	2.1 (1.9 ; 2.2)
**Multimorbidity excluding cancer**	Moderate Multimorbidity	1.2 (1.2 ; 1.3)	1.2 (1.2 ; 1.3)
	Severe Multimorbidity	1.6 (1.5 ; 1.6)	1.5 (1.4 ; 1.6)

Reference: No multimorbidity, Male, 65-75 years old, Cohabiting, Basic educational level, 0 previous hospitalisations previous year
Multimorbidity level: No 0-1 chronic condition, Moderate 2-3 chronic conditions, Severe ≥4 chronic conditions reported in the National Patient Register or via prescription for medications in the National Prescription Registry
*Variables included in the regression model: Age (group), sex, cohabitation status, educational status, previous hospitalisations

**Death within 90 days, N (%)**	2,415 (6)	6,703 (11)	10,468 (14)

Results from the analyses of the association between multimorbidity level and 30-day readmissions and 30-day mortality are shown in the table above.

Removing hypertension did not alter the effect estimates significantly on either the primary or secondary outcome. This indicates that hypertension is not a strong driver of the association and does not contribute uniquely to the association between multimorbidity and readmissions or mortality.
On the other hand, the removal of cancer from the index contributed to decrease in the HR of mortality from 1.6 (1.5 ; 1.8) to 1.2 (1.2 ; 1.3) for moderate multimorbidity, and likewise, from 2.2 (2.0 ; 2.4) to 1.5 (1.4 ; 1.6) for severe multimorbidity. The same pattern was evident in the effect estimates regarding 30-day readmissions. This suggests a significant role of cancer on both the association between multimorbidity and mortality and readmissions.
